# Follow-Up of New SSRI Prescriptions for Depression and Anxiety in Primary Care

**DOI:** 10.1192/bjo.2022.303

**Published:** 2022-06-20

**Authors:** Stephen Hughes

**Affiliations:** University of Nottingham, Nottingham, United Kingdom

## Abstract

**Aims:**

The aim of the audit was to review the follow-up of new SSRI prescriptions for anxiety and depression in a primary care setting and to evaluate this against relevant guidance, including that provided by NICE. NICE guidelines recommend initial follow-up for patients newly prescribed SSRIs for depression at either 1 week or 2 weeks dependent on patients age and the perceived risk of suicide or self-harm.

**Methods:**

An audit was carried out of new SSRI prescriptions and subsequent follow-up for 52 patients in a primary care practice in North Derbyshire covering the period January to August/September 2021.

The audit used patient notes which were manually reviewed to assess the initial consultation, prescription, documented suicide/self-harm risk assessment and follow-up plans. The length to initial follow-up and the number of subsequent follow-up appointments were also assessed.

**Results:**

The audit found that the median time to initial follow-up was 14.5 days for patients aged 18–30 years with only 12% compliant with the NICE recommendation of 1 week to follow-up. The median time to initial follow-up was 17.5 days for patients aged >30 years with only 19% compliant with the NICE recommendation of 2 weeks to follow-up. There were no significant differences in follow-up between males and females. 96% and 77% of initial consultations included a documented suicide risk assessment for patients aged 18–30 years and >30 years respectively. 88% of the new SSRI prescriptions were for sertraline 50 mg.

**Conclusion:**

The above findings were presented to the clinical team at the primary care practice meeting with reminders of the NICE recommendations for follow-up and how these vary between different patient groups. The practice will carry out a repeat audit in 12 months.

